# Supplementary material for: Autoreactivity of Broadly Neutralizing Influenza Human Antibodies to Human Tissues and Human Proteins
Source: Viruses. 2020 Oct 8;12(10):1140. doi: 10.3390/v12101140 (PMC7600923; doi:10.3390/v12101140)
Supplement: Supplementary file 1 [file viruses-12-01140-s001.pdf]

Table S1: List of host proteins reactive to influenza bNAbs in ProtoArrays

	Protein
1	N-acylaminoacyl-peptide hydrolase (APEH)
2	Enhancer of mRNA decapping 3 homolog (S. cerevisiae) (EDC3)
3	Serine/threonine/tyrosine interacting protein (STYX)
4	Tripartite motif-containing 21 (TRIM21)
5	Signal transducer and activator of transcription 4 (STAT4)
6	Mitogen-activated protein kinase 6 (MAPK6)
7	JO-1
8	Thyroglobulin

Table S2: Signal binding ratio of representative bNAbs relative to control 151K signal in IgG depleted human sera in ProtoArray.

PROTEIN	C6261	C8020	F10
STAT4	681.96	222.41	2.05
APEH	503.80	1.64	1.41
EDC3	221.14	2.41	1.22
TRIM21	2.37	2.37	2.19
BIRC8	4.66	122.51	1.65
STYX	2.70	182.46	1.63

Table S3: Competition of host proteins with Influenza HA binding to bNAbs

	C6261	CR8020	C9114	F10	FI6	C179	CH65
	%H1	%H3	%H1	%H1	%H1	%H1	%H1
**APEH**	100	100	99.3	100	100	99.7	98.9
**TRIM21**	100	99.2	100	100	100	96.3	100
**EDC3**	23.1	99.3	93.3	99.8	92.9	97.8	100
**STYX**	100	96.7	95.8	100	100	99.9	100
**STAT4**	100	100	98.1	100	91.6	89.6	90
**MAPK6**	100	93.9	97.2	100	100	92.6	94.5
**JO-1**	95.4	94.5	87.3	88.7	96.2	92.2	98.5
**Thyroglobulin**	99.2	100	93	100	99.6	94.4	94.9

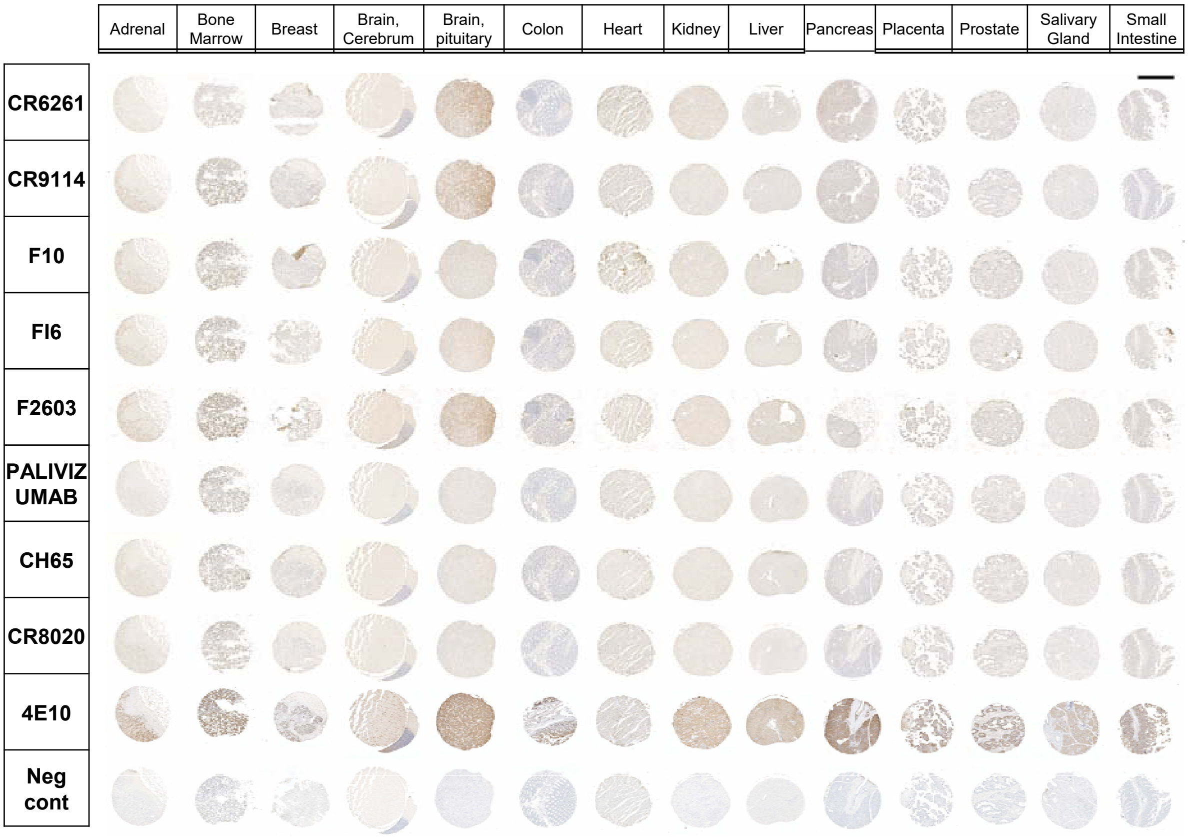

Supplementary figure 1. Representative binding images of each bNAb to normal human tissues. Scales indicates 1 mm.

```
          10        20        30        40        50        60
....|....|....|....|....|....|....|....|....|....|....|....|
MATDWLGSIVSINCGDSLGVYQGRVSAVDQVSQTISLTRPFHNGVKCLVPEVTFRAGDIT

          70        80        90       100       110       120
....|....|....|....|....|....|....|....|....|....|....|....|
ELKILEIPGPGDNQHFGDLHQTELGPSGAGCQVGINQNGTGKFVKKPASSSSAPQNIPKR

         130       140       150       160       170       180
....|....|....|....|....|....|....|....|....|....|....|....|
TDVKSQDVAVSPQQQQCSKSYVDRHMESLSQSKSFRRRHNSWSSSSRHPNQATPKKSGLK

         190       200       210       220       230       240
....|....|....|....|....|....|....|....|....|....|....|....|
NGQMKNKDDECFGDDIEEIPDTDFDFEGNLALFDKAAVFEEIDTYERRSGTRSRGIPNER

         250       260       270       280       290       300
....|....|....|....|....|....|....|....|....|....|....|....|
PTRYRHDENILESEPIVYRRIIVPHNVSKEFCTDSGLVVPSISYELHKKLLSVAEKHGLT

         310       320       330       340       350       360
....|....|....|....|....|....|....|....|....|....|....|....|
LERRLEMTGVCASQMALTLLGGPNRLNPKNVHQRPTVALLCGPHVKGAQGISCGRHLANH

         370       380       390       400       410       420
....|....|....|....|....|....|....|....|....|....|....|....|
DVQVILFLPNFVKMLESITNELSLFSKTQGQQVSSLKDLPTSPVDLVINCLDCPENVFLR

         430       440       450       460       470       480
....|....|....|....|....|....|....|....|....|....|....|....|
DQPWYKAAVAWANQNRAPVLSIDPPVHEVEQGIDAKWSLALGLPLPLGEHAGRIYLCDIG

         490       500
....|....|....|....|....|...
IPQQVFQEVGINYHSPFGCKFVIPLHSA
```

Supplementary figure 2: Sequence of human EDC3 with residues highlighted that potentially mimic contact site for HA binding with bNAb CR6261. The residues shaded in yellow, cyan and green are shown in similar colors in Figure 1D.
